# Clinical Utility of Droplet Digital PCR to Monitor BCR-ABL1 Transcripts of Patients With Philadelphia Chromosome–Positive Acute Lymphoblastic Leukemia Post-chimeric Antigen Receptor19/22 T-Cell Cocktail Therapy

**DOI:** 10.3389/fonc.2021.646499

**Published:** 2021-04-07

**Authors:** Yuqi Guan, Meilan Zhang, Wei Zhang, Jiachen Wang, Kefeng Shen, Kai Zhang, Li Yang, Liang Huang, Na Wang, Min Xiao, Jianfeng Zhou

**Affiliations:** ^1^Department of Hematology, Tongji Hospital, Tongji Medical College, Huazhong University of Science and Technology, Wuhan, China; ^2^PerfectGen, Zhuhai, China

**Keywords:** droplet digital PCR, CAR19/22 T-cell cocktail, minimal residual disease, relapsed/refractory, acute lymphoblastic leukemia, Philadelphia chromosome positive, BCR-ABL1

## Abstract

Philadelphia chromosome–positive acute lymphoblastic leukemia (Ph^+^ ALL) accounts for 20–30% of adult patients with ALL, characterized by translocation of *t*_(9, 22)_. Tyrosine kinase inhibitors (TKIs) have significantly improved the outcome even though there are still some problems including relapse due to drug-resistant mutations and suboptimal molecular remission depth. Previously, we reported the safety and efficacy of sequential infusion of CD19/22 chimeric antigen receptor T-cell (CAR-T) immunotherapy in the treatment of relapsed/refractory (R/R) B-cell neoplasms including cases with Ph^+^ ALL. Given possible deeper reaction, more patients were expected to reach optimal minimal residual disease (MRD) response. An alternative method, duplex droplet digital PCR (ddPCR) with high sensitivity was established, which could provide absolute quantification of MRD without the need for calibration curves. Here, we retrospectively collected 95 bone marrow samples from 10 patients with R/R Ph^+^, who received 19/22 CAR-T-cell cocktail therapy. Notably, sequential molecular remission for more than 3 months (SMR3), a significant indicator based on ddPCR after CAR-T infusion was established, which was defined as a sequential molecular remission for not <3 months with negative MRD. In this cohort, no recurrence was observed in six patients achieving SMR3, where four of whom accepted allogeneic hematopoietic stem cell transplantation (allo-HSCT) after CAR-T cell regimen. Unfortunately, the other four patients who did not reach SMR3 relapsed, and did not receive extra specific treatment except CAR-T regimen. To sum up, ddPCR may be an alternative, especially when nucleic acid was insufficient in clinical practice. No achievement of SMR3 may be an early warning of potential relapse after CAR-T and indicating the initiation of other therapies including allo-HSCT.

## Introduction

Acute lymphoblastic leukemia (ALL) is one of the most common childhood leukemia (1, 2). Ph chromosome derived from the reciprocal translocation of *t*(9, 22) (q34; q11.2), leading to the expression of chimeric BCR-ABL1 gene, accounts for more than 20–30% of all adult cases with ALL. The incidence of Ph^+^ ALL increases with age and that of B-precursor patients with ALL who are older than 60 reaching 50% (3, 4). The Ph chromosome and the BCR-ABL1 fusion gene were historically associated with a dismal prognosis, particularly in the absence of allo-HSCT (5, 6). Over recent years, tyrosine kinase inhibitors (TKIs) has revolutionized the treatment of Ph^+^ ALL by affecting the tyrosine kinase activity in the transformation of cells (7). More than 80% of cases could achieve CR with a 5-year overall survival (OS) rate up to 30–40% (8, 9). While the challenges that still need to be addressed including relapse due to drug-resistant mutations and suboptimal molecular remission depth in order to improve patient outcomes. Over the last few years, chimeric antigen receptor T-cell (CAR-T) therapy has emerged as a promising new therapeutic approach, which has robust activity against relapsed/refractory (R/R) B-cell lineage ALL (10–12). The potential effectiveness of CD19/22 CAR-T-cell in treating R/R B-cell neoplasms, including such high-risk genetic or chromosome aberrations as Ph^+^ ALL, has been demonstrated by our center previously (13). The investigation of residual BCR-ABL1 transcriptional levels shortly after starting TKI represent the molecular marker for the evaluation of Ph^+^ ALL, defining the depth of molecular remission, and suggesting clinical decisions (14–16). Given deeper reaction demonstrated by CAR-T, it is expected that more patients can reach complete molecular response. Quantitative real-time PCR (qPCR) methods are routinely used to monitor BCR-ABL1 transcript levels in patients with Ph^+^ ALL (17). While qPCR involves separate measurements of target and reference DNAs, it requires standard curves, and is susceptible to PCR inhibition. The droplet digital (ddPCR) is a burgeoning sensitive molecular technique to realize absolute quantification, which divides the reaction system into a mass of reaction units using a water–oil emulsion droplet system and gets PCR data with Poisson statistics (10, 11). It was found to be able to detect very low BCR-ABL1 levels (10^−5^ and below) using ddPCR (18–20). Some studies previously reported that when testing MRD <10^−4^, the accuracy of ddPCR test results measures even higher than qPCR (21). In general, an optimized duplex ddPCR, with high sensitivity, could provide absolute quantification without the need for standard curves. Factors affecting chimeric antigen receptor T cell (CAR-T) are complicated that it is difficult to predict the persistence of CAR-T effects with a single indicator to make choices about whether and when to bridge patients to allogeneic hematopoietic stem cell transplantation (allo-HSCT) (22). A proper index may be helpful to find out candidates who are in the risk of cancer recurrence after CAR-T and indicate timely therapy selection. A trend in BCR-ABL1 transcript reduction has been supposed to be much more informative than a single value in TKI minimal residual disease (MRD) monitoring. Therefore, we focused on the trend of BCR-ABL1 transcripts post-CAR-T infusion using ddPCR. We retrospectively identified 10 patients with Ph^+^ who had prospective bone marrow samples and clinical data collection. The clinical utility of ddPCR was explored in MRD detection, and the indicator of possible relapse was assumed based on ddPCR.

## Materials and Methods

All cases in this study were collected from the CD19/22 sequential CAR-T clinical trial with Chinese Clinical Trial Registry (ChiCTR OPN 16008526) in our center. The investigations were approved by Tongji Hospital, Tongji Medical College, Huazhong University of Science and Technology. Eligible patients from here were R/R to multi-line treatments including chemotherapy agents combined with TKIs. Accordingly, patients were diagnosed according to the classification of hematopoietic and lymphoid tissue tumors by the WHO. All of them were in good performance status, with measurable disease and a life expectancy of 12 weeks or more before the clinical trial of CAR-T infusion, but without uncontrollable infection, evident neurological lesions, or active graft vs. host disease (GVHD). More details about the study design and criteria of the clinical trials conducted in our center have been described previously (13). Written informed consent was obtained from each patient. After taking fludarabine and cyclophosphamide orally for 3 days, patients were infused a total of 2 ~ 4 × 10^6^/kg CD19 CAR-T cells, followed by 2 ~ 4 × 10^6^/kg CD22 CAR-T cells with an interval of several days generally. Schematic diagrams of anti-CD19 CAR-T and anti-CD22 CAR-T have been shown in Supplementary Figure 1. Patients enrolled in the CAR-T clinical trials underwent bone marrow biopsies once per month for the first 6 months, followed by once every 3 months for the remainder of the study. The remaining samples after clinical testing were preserved under appropriate conditions (MRD) monitoring by ddPCR and qPCR were carried out using the above remaining samples, which have the same frequency. Subsequently, four patients with relapse after CAR-T therapy were bridged to allo-HSCT. Expression of CD19 and CD22 was confirmed by using flow cytometry. Evaluations of response to treatment were based on the guidelines of National Comprehensive Cancer Network (NCCN). Cytogenetic and genomic aberrations were identified by karyotyping, qPCR, fluorescence *in situ* hybridization (FISH), and next-generation targeted sequencing. *In vivo* expansion of CD19- and CD22-T cells were measured by ddPCR as we had previously reported (23). K562 cells (transcript positive samples), BA/F3 cells (wild-type samples), samples from patients with Ph^+^, and normal RNA samples from healthy donors were used to determine the performance of ddPCR.

### Evaluation of BCR-ABL1 Transcripts

To define the limit of detection for the P210 (e14a2) transcript, a serial dilution was performed using cDNA from K562 cell lines and wild-type background BA/F3 cells, with the following concentrations: 100, 50, 10, 1, 0.032, 0.01, and 0.001%. Samples from patients with Ph^+^(e1a2/e13a2) and normal RNA samples from healthy donors were mixed to generate a single samples such as P190(e1a2)-pool, P210(e13a2)-pool, and diluent-pool, respectively. Similarly, the limits of detection for the BCR-ABL1 e1a2 and e13a2 assays were defined by the P190(e1a2)-pool and P210(e13a2)-pool serial dilutions. RNA was extracted from bone marrow specimen using the QIAamp RNA Blood Kit (QIAGEN company, Hilden, Germany) and were reverse transcribed to cDNA using a standardized protocol (24). A standard volume of 20 μl of reaction mix with 2 μl of DNA template was conducted in ddPCR using the Quantalife QX200 ddPCR system (Bio-Rad Laboratories, CA, USA) in the following conditions: 95°C for 5 min, 95°C for 30 s, 60°C for 1 min (40 cycles) with a 10 min hold at the temperature of 98°C, and a final hold at 4°C. All assessments of clinical samples using ddPCR were repeated in three duplicates. Diluent-pool, as the negative control, was tested in 10 replicates to identify the cut-off value. ABL1 was selected as the control gene to compensate for variations in the quality and quantity of RNA and cDNA. Results were considered to be effective when the number of droplets per well was at least 10,000 and the correct value was provided by the housekeeping gene. The results were analyzed using QuantaSoft software version 1.7.4 (Bio-Rad Laboratories, CA, USA), strictly according to the recommendations of the manufacturer. The classical qPCR monitoring was performed relatively following the instruction of the manufacturer instructions on ABI Step One Plus™ Real-Time PCR System (Applied Biosystems, USA). A laboratory-specific correction factor (CF) was used to convert the ratio of %BCR-ABL1/ABL1 (P210 transcript) to international scale (IS) (% IS = Ratio × 100 × CF).

### Statistical Analyses

The comparison of BCR-ABL1 transcript levels between qPCR and ddPCR acquired from clinical samples was performed using the Spearman's rank-order correlation coefficient analysis. Bland–Altman analysis was used to evaluate the agreement of results in clinical samples of these two methods mentioned above. Subsequently, a comparison of BCR-ABL1 transcript levels medians measured by both methods was examined using the Mann–Whitney test. A *p* < 0.05 was considered significant statistically. The relapse was confirmed as per the guidelines of NCCN (25). The cumulative incidence of relapse (CIR) was estimated by means of the Kaplan–Meier method and was compared with the use of the log-rank test. GraphPad Prism 7 was used in the statistical analysis.

## Results

A total of 10 patients (median age, 41.3 years; minimum, 28 years; maximum, 51 years; 7 men and 3 women) with Ph^+^ ALL were included, all of whom were received CD19/22 cocktail CAR-T therapy. The morphologic diagnosis was confirmed in all cases by qPCR, karyotypic analysis, and FISH, as previously reported. Half of them had a history of Ponatinib usage, including the patients, P1, P2, P6, P9, and P10. The median follow-up duration for the entire cohort was 472 days (min: 246 days; max: 921 days). Only one patient died 265 days after CAR-T (P2) as a result of high tumor burden from disease recurrence (**Figure 2**, Table 1). The main characteristics of the patients are also reported in Table 1.

**Table 1:** Characteristics of 10 patients with relapsed/refractory (R/R) Ph^+^ who accepted the chimeric antigen receptor (CAR) 19/22 T-cell cocktail therapy.

**No**.	**G**	**Age**	**Transcript**	**Karyotype**	**ABL1 resistance mutation**	**Prior usage of TKIs**	**Before CAR-T**		**Dose of CAR-T** **× 10^**6**^/**kg****		**Best response (ddPCR)**	**Subsequent allo-HSCT**	**Days to bridge into HSCT**	**Follow-up days**	**Status of last follow-up**
							**BM tumor burden %**	**WBC count × 10^9^/l**	**CD19**	**CD22**					
P1	M	30	P210	Complex	T315I	I, D, P	14.15	11.31	3	2	Negative	No	–	246	Relapse
P2	M	42	P190	Complex	T315I	I, D	11	5.63	2	2	Negative	No	–	265	*Relapse*^(*dead*)^
P3	M	45	P190	Complex	–	D	84	29.34	1	5.8	<10^−4^	No	–	442	Relapse
P4	M	48	P210	Complex	–	D	40.26	17.56	6.63	7.78	Negative	No	–	268	Relapse
P5	F	36	P210	Complex	T315I	P	90.10	100.27	4	4	<10^−3^	Yes	210	750	CR
P6	M	46	P210	Complex	T315I	I, D, P	23.30	14.58	4	2	<10^−3^	Yes	178	921	CR
P7	F	45	P210	Complex	T315I	D, P	40	2.36	4	2	Negative	Yes	120	847	CR
P8	F	28	P190	Complex	–	D	74.80	7.08	6	6	Negative	Yes	60	350	CR
P9	M	42	P190	Complex	–	D, P	95	10.59	2	2	Negative	No	–	285	CR
P10	M	51	P190	Complex	–	D, P	64	16.05	2	2.32	Negative	No	–	351	CR

*CAR19 and CAR22 T cells were infused separately on successive days from day zero as reported previously. Prior TKIs usage: I, imatininb; D, dasatinib; P, ponatinib; No., number; CR, complete response; WBC, white blood cell; allo-HSCT, allogenic hematopoiesis stem cell transplant*.

**Figure 1:**
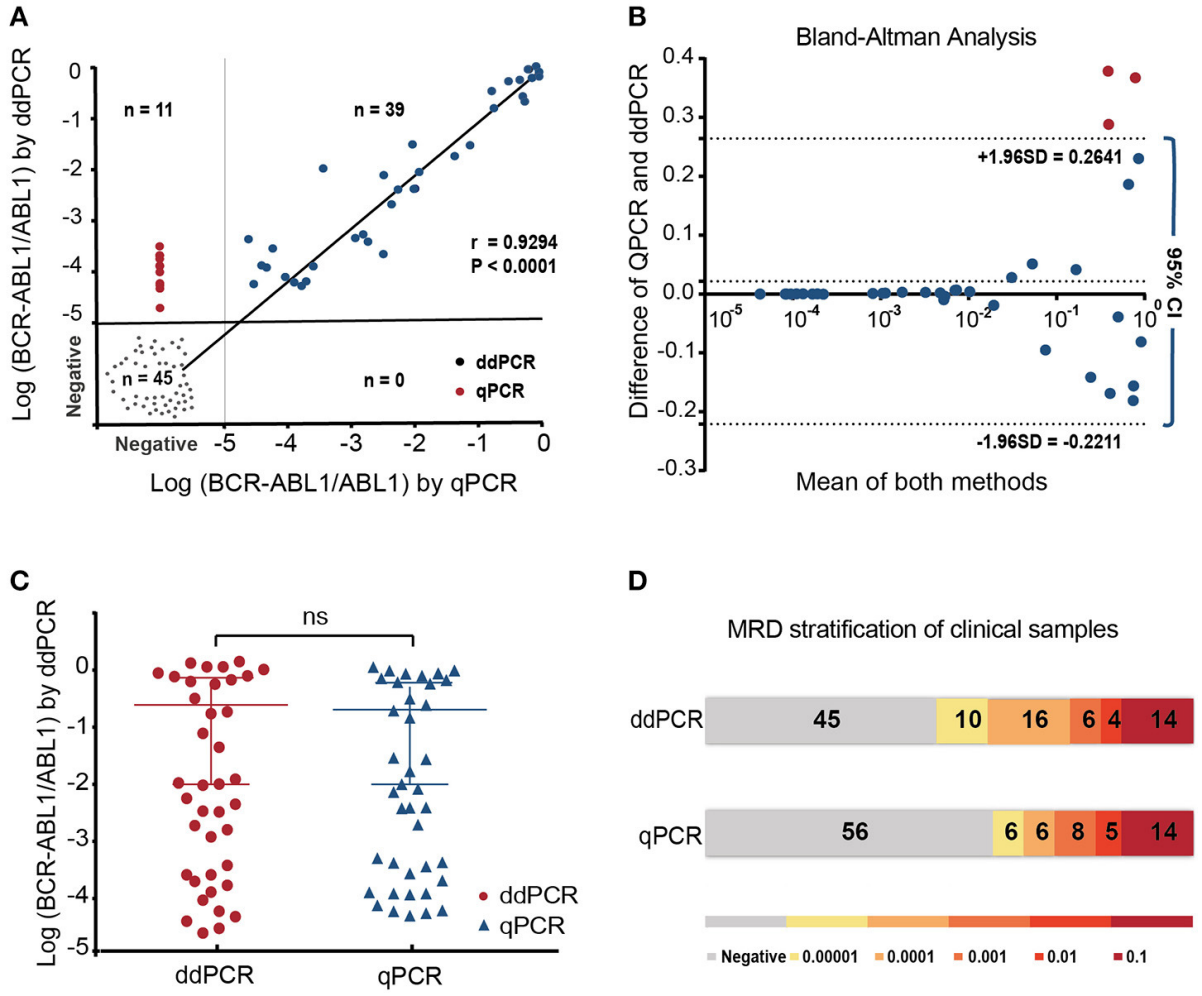
The performance of ddPCR and qPCR in minimal residual disease (MRD) analysis of 95 follow-up samples from 10 patients with Ph^+^ ALL post-chimeric antigen receptor T cell (CAR-T). **(A)** MRD results were highly correlated between the two platforms using Spearman's test (*r* = 0.9257; *p* < 0.0001) and showed good linearity on samples over the detection range and good linearity (*p* = 0.0042); **(B,C)** Bland–Altman analysis and paired *t*-test showed the reliable correlation of two methods **(D)**. All positive quantitative PCR (qPCR) results were identified as positive by droplet digital PCR (ddPCRP). Among 56 samples found negative using qPCR, 11 were detected positive using ddPCR, which indicated higher sensitivity of ddPCR.

Data from standard serial dilutions showed remarkable linearity, reliability, and a precision of up to 0.001% by ddPCR (Supplementary Figure 2, Supplementary Table 1). The results would be considered as negative, when no positive droplets were found, since no background from negative samples was detected. When duplicates showed inconsistency, the results would be re-interpreted. The ddPCR and qPCR experiments were successfully performed in a total of 95 samples during the follow-up. ALL samples identified positive by qPCR also showed positive by ddPCR analysis (Figures 1A,D). MRD results were highly correlated between the two platforms using Spearman's test (*r* = 0.9257; *p* < 0.0001) and showed good linearity on samples over the detection range and good linearity (*p* = 0.0042; Figures 1B,C). Agreement between the two methods was further assessed using a Bland–Altman plot. The mean bias was 0.02152 with 95% limits of agreement ranging from −0.2211 to 0.2641 indicating that there was no systematic difference between the two methods.

**Figure 2:**
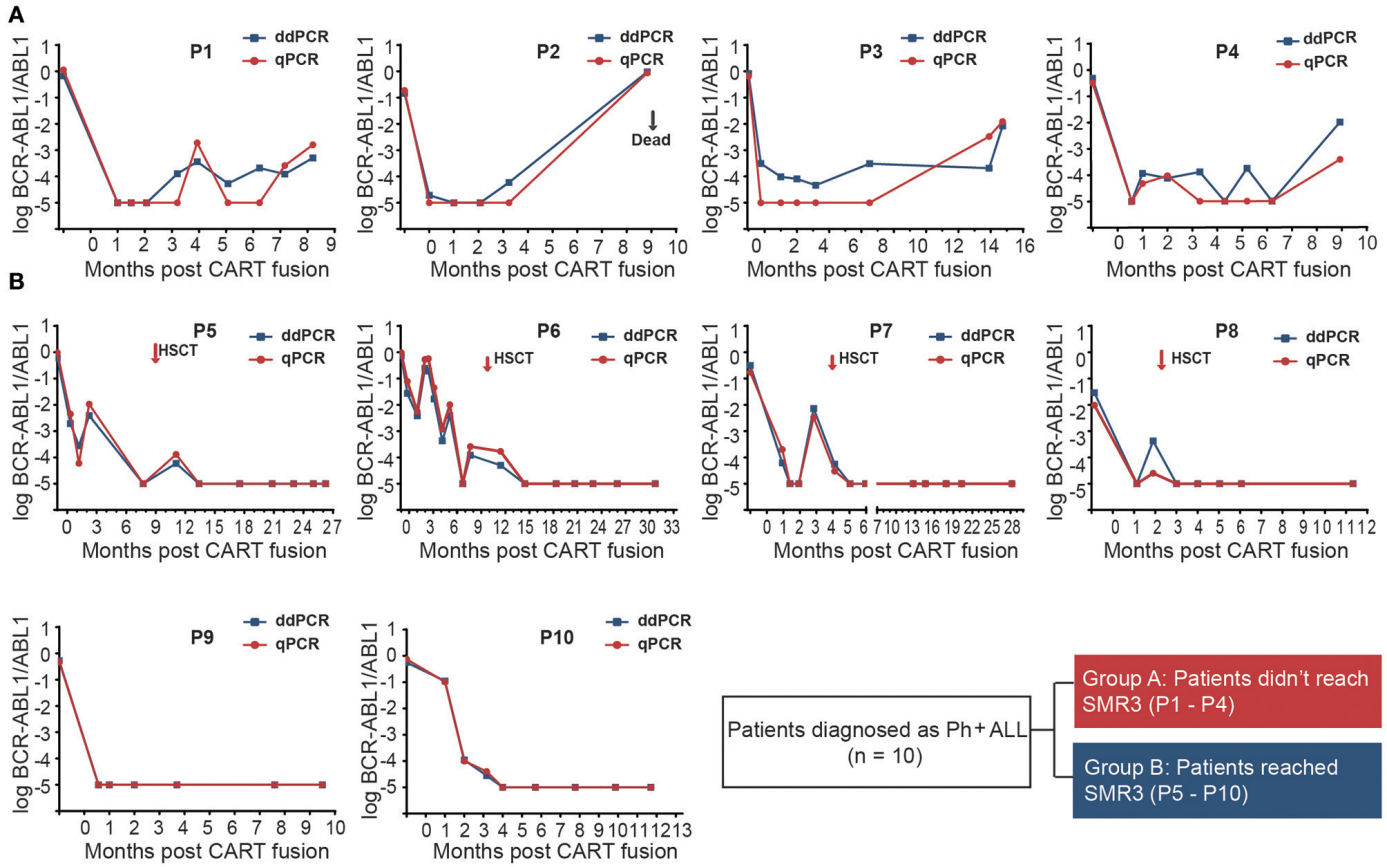
Periodic MRD monitoring of 10 patients with chimeric antigen receptor T-cell (CAR-T) therapy and with Ph^+^ ALL by ddPCR and qPCR. The x-axis represents the follow-up time from the initial days of CAR-T infusion and the y-axis represents log (BCR-ABL/ABL). The result <10^−5^ was defined as negative. **(A)** Patients (P1–P4) without SMR3 relapsed when they received no extra-specific treatment except CAR-T regimen. It was noted that ddPCR could identify positive recurrence even several months before qPCR testing in patient P3; **(B)** Patients with the SMR3 indicator could maintain CR for longer time. The cases such as P9 and P10 showed optimal response to CD19/22 therapy. While in patients (P5–P8) with unsteady BCR-ABL1 transcripts after CAR-T, a better remission may be brought to them in the wake of allo-HSCT.

**Figure 3:**
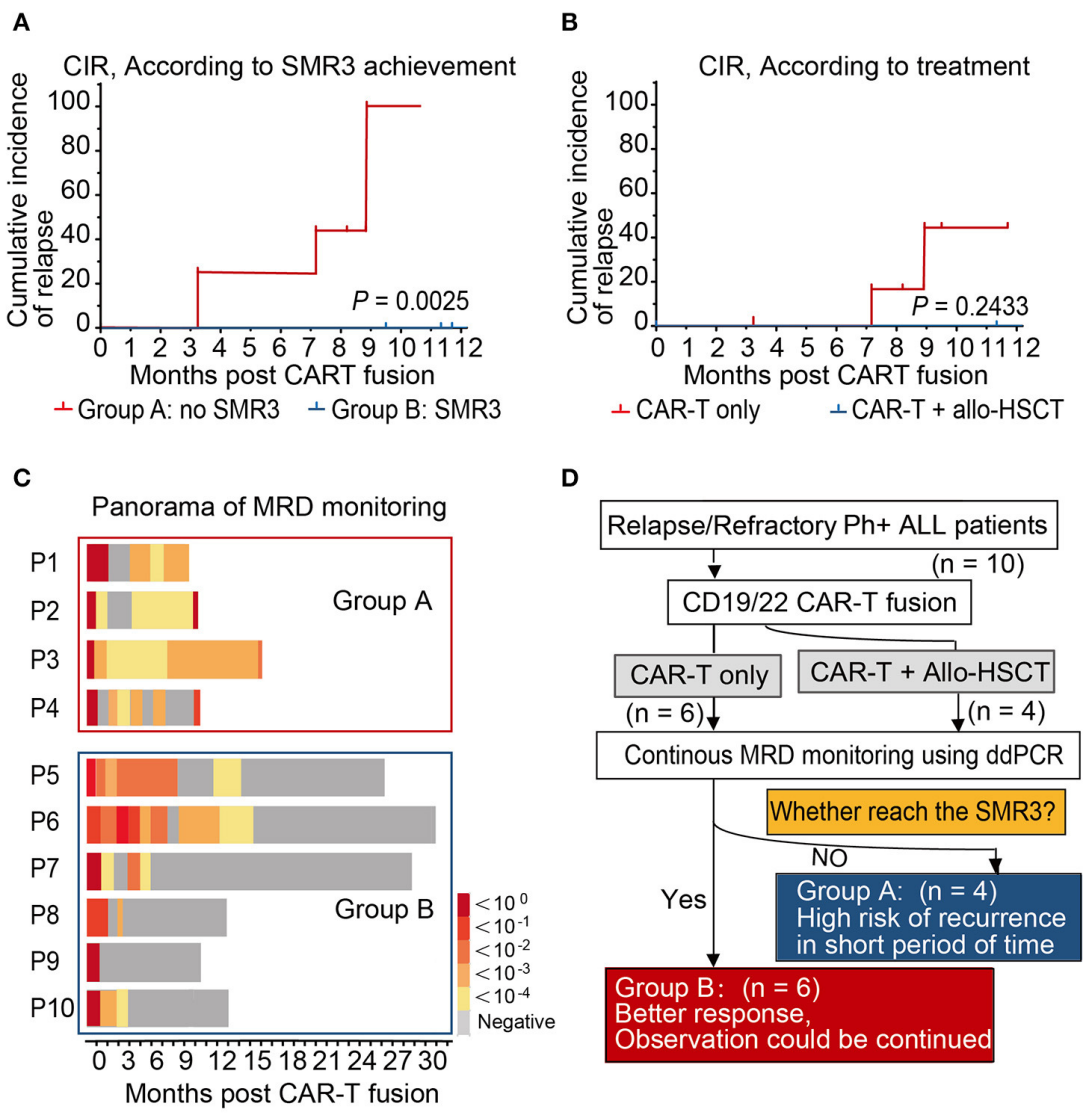
Prognosis and panorama of MRD monitoring of patients. **(A,B)** The 1-year cumulative incidences of relapse of patients according to SMR3 definition and treatment. A better prognosis may be expected. **(C)** The full view of MRD monitoring in Group A showed a persistent negative in the follow-up, which supported the prediction efficacy of SMR3 model. **(D)** Patients with Ph^+^ post-chimeric antigen receptor T-cell (CAR-T) therapy were assigned to different groups based on the sequential molecular remission for more than 3 months (SMR3) signature so as to predict the corresponding prognosis. When reaching the definition of SMR3, patients were expected to have better prognosis. However, no achievement of SMR3 may be an early warning of potential relapse and indicating the initiation of other therapies including allo-HSCT.

BCR-ABL1 transcript levels of all patients showed a decline after CAR-T infusion. The dynamic monitoring of all patients on BCR-ABL1 transcripts by ddPCR and qPCR is shown in Figure 2). SMR3, a significant indicator based on ddPCR after 19/22 CAR-T cell therapy was established, which was defined as a sequential molecular remission for not <3 months with negative MRD results by ddPCR. Patients were divided into two groups based on the achievement of the definition of sequential molecular remission for more than 3 months (SMR3), regardless of subsequent treatment. In this cohort, six patients who achieved SMR3 had no recurrence, including four patients who accepted HCST after CAR-T regimen. On the contrary, the other four patients who did not reach SMR3 were found relapsed; unfortunately, all of whom had obtained no extra specific treatment except CAR-T only regimen. The CIR was not consistent in the other subgroups according to the treatment after CAR-T and the CIR of patients (*n* = 6) with CAR-T only at month 12 was 43.75%. We found that none of the patients who took subsequent allo-HSCT showed relapse during the follow-up period (Figures 3A,B and Table 1). Panorama of MRD monitoring of patients is shown in Figure 3C. Patients with Ph^+^ post-CAR-T were assigned to different groups based on the SMR3 signature to predict the corresponding prognosis. When reaching the definition of SMR3, patients were expected to exhibit a better prognosis. No achievement of SMR3 may be an early warning of potential relapse and the initiation of other therapies including allo-HSCT in this study (Figure 3D).

## Discussion

It is for the first time that our study applied a molecular strategy to explore the periodic assessments of BCR-ABL1 transcripts in patients with Ph^+^ B-ALL post-CD19/22 CAR-T therapy using ddPCR. This method exhibited high sensitivity and agreement on samples over the detection limitation, compared r to classical qPCR methods. Despite similar sensitivity, the ddPCR can realize absolute quantitation without the need for standard curves, therefore offering the possibility of inter-laboratory reproducibility and to detect low levels of MRD, thereby giving a better distinction of patients from MR4 and MR4.5 (26). The sufficient amount of RNA input may be necessary to improve the analytical sensitivity of BCR-ABL testing. More indexes of ddPCR methodology, including IS value still need to be identified. Recently, no published guidelines for ddPCR data interpretation have been recommended for monitoring Ph^+^ ALL. Alternatively, it was demonstrated that ddPCR may be a promising methodology for MRD monitoring. Moreover, a predictive indicator, such as SMR3, was put forward, which was defined as a sequential molecular remission for not <3 months with negative MRD results by ddPCR. When patients could reach the definition of SMR3 within half a year after CAR-T infusion, a better prognosis is expected.

This study represents the application of ddPCR in MRD monitor on samples from patients with R/R Ph^+^ ALL after CAR-T. It was shown in our clinical data that all positive samples detected by qPCR were confirmed to be positive by ddPCR that shows the reliability of ddPCR in MRD detection based on established protocols (Figures 1A,D). It is worth noting that there were few low burden samples found positive only when using ddPCR in our study (Figures 1A,D). A case in point is the patient (P3) in Figure 2A, who was of low risk according to the qPCR, but was predicted of high risk of relapse from the very beginning post-CAR-T therapy by the ddPCR monitoring (Figure 2A). It was acknowledged that ddPCR uses a water–oil emulsion droplet system to divide the reaction system into a large number of reaction units, and can eliminate its reliance on the standard curve. Therefore, ddPCR may be an alternative in current MRD monitoring when nucleic acid obtained from bone marrow samples was insufficient in clinical practice. More comparisons will have to be carried out in further study. It has been approved that the use of CD19 CAR-T-cell therapy in treating patients (age <26 years) with R/R precursor B-cell ALL by FDA. CAR-T cells therapy have shown significantly better prognosis than traditional regimens (27). Although the third generation TKI, such as Ponatinib, was designed to overcome the T315I mutation, compound mutations, and some other mechanisms, such as BCR-ABL1 independent factors may also impact the clinical response to Ponatinib (28). It has been reported that this new option could exert profound cytotoxicity against Ph^+^ ALL cell lines and that its killing effects do not impart by resistance mutations in BCR-ABL1-kinase domain (29). Therefore, CAR-T regimen may be sometimes an alternative solution to TKI drug resistance. However, the lasting therapeutic effect of CD19-directed therapies may also be challenged by the loss of epitope or disrupted CD19 membrane trafficking (30, 31). Meanwhile, CD22 retains its expression on the surface of leukemia cells (32, 33). There has been speculation that an optimal property could be expected in dual-targeting of CD19 and CD22 CAR-T. The real-time response and clinical efficacy of CAR-T therapy were dynamically recorded by ddPCR. In this study, the promising immunotherapy was demonstrated by the significant decreasing tendency of the BCR-ABL1 transcript levels after infusion of CD19/22 CAR-T cells in all cases (Figure 2A). Although the MRD-negative CR rates post-CAR T-cell therapy are impressive in patients with Ph^+^ ALL, reports on the durability of responses are limited. The duration of remissions may be associated with the expansion and persistence of CAR-T cell *in vivo* and some other clinical features (22, 34). It is of great importance to find a sensitive and predictive index to identify patients at the risk of relapse. There have been several researchers who explored the reliability of ddPCR in MRD monitoring of other hematologic malignancies (35, 36). Many studies reported some comparisons in monitoring the BCR-ABL1 fusion transcript by ddPCR and qPCR in patients with chronic myeloid leukemia (18, 37). Nowadays, periodic MRD assessment after treatment recommended by the guideline of NCCN for the MRD level is of significant predictive value for the risk of relapse and prognosis, as well as for later treatment strategy (2, 14, 38). PCR analysis of rearranged immunoglobulin heavy chain (IgH) and T-cell receptor (TCR) genes are recommended for MRD monitoring as per the guideline of NCCN, while the classical allele-specific oligonucleotide (ASO)-PCR is both time-consuming and labor-intensive and requires extensive knowledge. Recently, next-generation sequencing (NGS)-based IGH or TCR-clonal rearrangements have been introduced (39), which expand the sensitivity of MRD detection from 1 blast cell in 10^4^ to 10^5^ cells offered by PCR to 1 in 10^7^ cells. It has been shown to be predictive of relapse in children with B-ALL receiving standard chemotherapy with this method (40). However, it may not be used widely used due to disproportional target of the super-multiplex PCR, difficult differentiation from normal clonal background, and unclear definition for positivity. A complete molecular response at 3 months after a TKI is considered as a strong prognostic factor and indicating a rapid change of therapy before relapse (2, 15, 41, 42). Moreover, it has been recognized that a trend in BCR-ABL1 transcript reduction is indeed much more informative than as a single value: the kinetics of BCR-ABL1 transcripts during the first 3 months has thus been proposed as a more reliable indicator of the ensuing molecular response and outcome (43–45). Similarly, we try to define a possible predictive indicator of post-CAR-T follow-up evaluation, targeting the kinetics of BCR-ABL1 fusion, in cases with Ph^+^ ALL. The NCCN guidelines recommend patients with undetectable levels of molecular remission for periodic MRD assessments (not more than every 3 months) (25). Some researchers have defined the sustained molecular CR as negative BCR-ABL transcript lasting for at least 3 months (46). It was shown in our study that the patients (P1, P2) soon reached a temporary molecular remission for 1–2 months relapse, while those who reached SMR3 (P5–P10) (sequential molecular remission for not <3 months) may have a more enduring remission (Figure 2). Based on the foregoing study, we put forward an indicator, SMR3, which was defined as the sequential molecular remission for not <3 months with negative results from the periodic monitoring of MRD using ddPCR post-CAR-T. When with the SMR3 signature, the patients may have better prognosis and observation, and may be continued under routine monitor, while for those could not reach the SMR3, relapse may be on the way. Bone marrow transplantation is considered as the cure for R/R ALL, while many patients are not eligible for transplant due to age or status of the disease. Some researchers thought of pretreatment with CAR-T cells as a bridge to allo-HSCT, which might give those patients with R/R ALL chances to have a CR and eligible to receive transplantation (47, 48). Here, we concluded a routine for the management of patients after CAR-T based on the SMR3 index (Figure 3D). If the patients could reach SMR3 after CAR-T infusion, better response may be expected and observation could be continued if they were not suitable or worried about the considerable transplantation-related morbidity and mortality. As for the patients with CAR-T, who failed to achieve SMR3, even in clinical remission status, they were susceptible to short-term recurrence and should be given timely treatment, such as early allo-HSCT. Considering the limited number of cases in this single-center retrospective clinical study, the ddPCR-based index of SMR3 still need to be explored and justified in the larger cohort.

Taken together, we found that the ongoing monitoring of BCR-ABL1 transcripts using ddPCR was a reliable approach to monitor MRD of patients with Ph^+^ ALL who have absolute quantitation and great applicability. We established an indicator, SMR3, based on the trends of periodic MRD assessment using ddPCR for the first time, which may play a role in the efficacy, evaluation, and relapse prediction for patients with Ph^+^ ALL post-CAR-T infusion. In spite of insufficient sample size, this study provides some hints for better recognizing patients at high risk and permitting pre-emptive intervention before relapse after CAR-T. Further study with larger samples will be conducted.

## Data Availability Statement

The original contributions presented in the study are included in the article/Supplementary Material, further inquiries can be directed to the corresponding author/s.

## Ethics Statement

The studies involving human participants were reviewed and approved by Tongji Hospital, Tongji Medical College, and Huazhong University of Science and Technology. The patients/participants provided their written informed consent to participate in this study.

## Author Contributions

YG made contribution to conceptualization, methodology, software, and visualization, and wrote the original draft. MZ collected samples, performed the sequence, analyzed the data, and validated the results. KZ gave the instrumental guidance and technique support. WZ, JW, KS, LY, LH, and NW collected samples and data. MX designed and supervised the study and including funding the acquisition. JZ supervised and reviewed the article. All authors contributed to the article and approved the submitted version.

## Conflict of Interest

KZ was employed by the company PerfectGen, Zhuhai, Guangdong Province, China. The remaining authors declare that the research was conducted in the absence of any commercial or financial relationships that could be construed as a potential conflict of interest.

## Supplementary Material

Supplementary Figure 1 Schematic diagram of anti-CD19 CAR-T and anti-CD22 CAR-T. The third generation CAR-T consists of a single-chain variable fragment against CD19 or CD22, two costimulatory domains from CD28 and 4-1BB, and an activation domain (CD3 zeta chain). SP, signal peptide; VL, variable chain; L, linker; VH, variable H chain.

Supplementary Figure 2 Performance of BCR-ABL1 droplet digital PCR (ddPCR) with different BCR-ABL1 fusion forms (e14a2, e13a2, and e2a1) by serial dilutions. **(A)** BCR-ABL1 P210 (e14a2) ddPCR assay limit-of detection determination by serial K562 cell dilutions; **(B)** BCR-ABL1 P210 (e13a2) assay by serial P210-pool dilutions; **(C)** BCR-ABL1 P190 (e2a1) assay by serial dilutions of P190-pool. The good linearity of transformations of BCR-ABL1 copies/ABL1 and of target values. *R*^2^ ≈ 1 indicates a remarkable correlation and no concentration-dependent bias.

Supplementary Table 1 Analytical performance parameters of the ddPCR assay. **(A)** Performance Parameters of the ddPCR P210 (e14a2) Assay by K562 cell lines dilution. **(B)** Performance Parameters of the ddPCR P210 (e13a2) Assay by P210 (e13a2)-pool. **(C)** Performance Parameters of the ddPCR P190 (e1a2) Assay by P190 (e1a2)-pool. cDNAs from pretreatment samples pooled [P210(e)-pool, P190 (e1a2)-pool] together with K562 cell lines were diluted in total cDNA from healthy donors (diluent-pool). ddPCR, droplet digital PCR. ddPCR data were analyzed with QuantaSoft analysis software. The target concentration in each sample was expressed as BCR-ABL1 copies/μg.

Supplementary Table 2 Baseline characteristics of all enrolled patients with Ph^+^.

Supplementary Table 3 Balanced characteristics of the patients with sequential molecular remission for more than 3 months (SMR3) or not.

## Abbreviations

R/R ALL: Relapsed/refractory acute lymphoblastic leukemia
CAR-T: Chimeric antigen receptor T cell
MRD: Minimal residual disease
ddPCR: droplet digital PCR
QPCR: The quantitative polymerase chain reaction
Allo-HSCT: Allogeneic hematopoietic stem cell transplantation
GVHD: graft vs. host disease
SMR3: sequential molecular remission for more than 3 months
OS: Overall survival
Ph: Philadelphia chromosome
CIR: Cumulative incidence of relapse.
