# Supplementary figures and images for: Clinical Utility of Droplet Digital PCR to Monitor BCR-ABL1 Transcripts of Patients With Philadelphia Chromosome–Positive Acute Lymphoblastic Leukemia Post-chimeric Antigen Receptor19/22 T-Cell Cocktail Therapy
Source: Front Oncol. 2021 Apr 7;11:646499. doi: 10.3389/fonc.2021.646499 (PMC8059437; doi:10.3389/fonc.2021.646499)

Supplementary Figure S1.

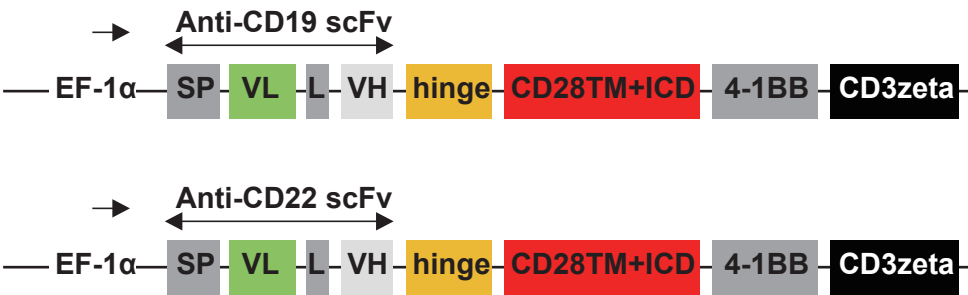

Supplementary Figure S2.

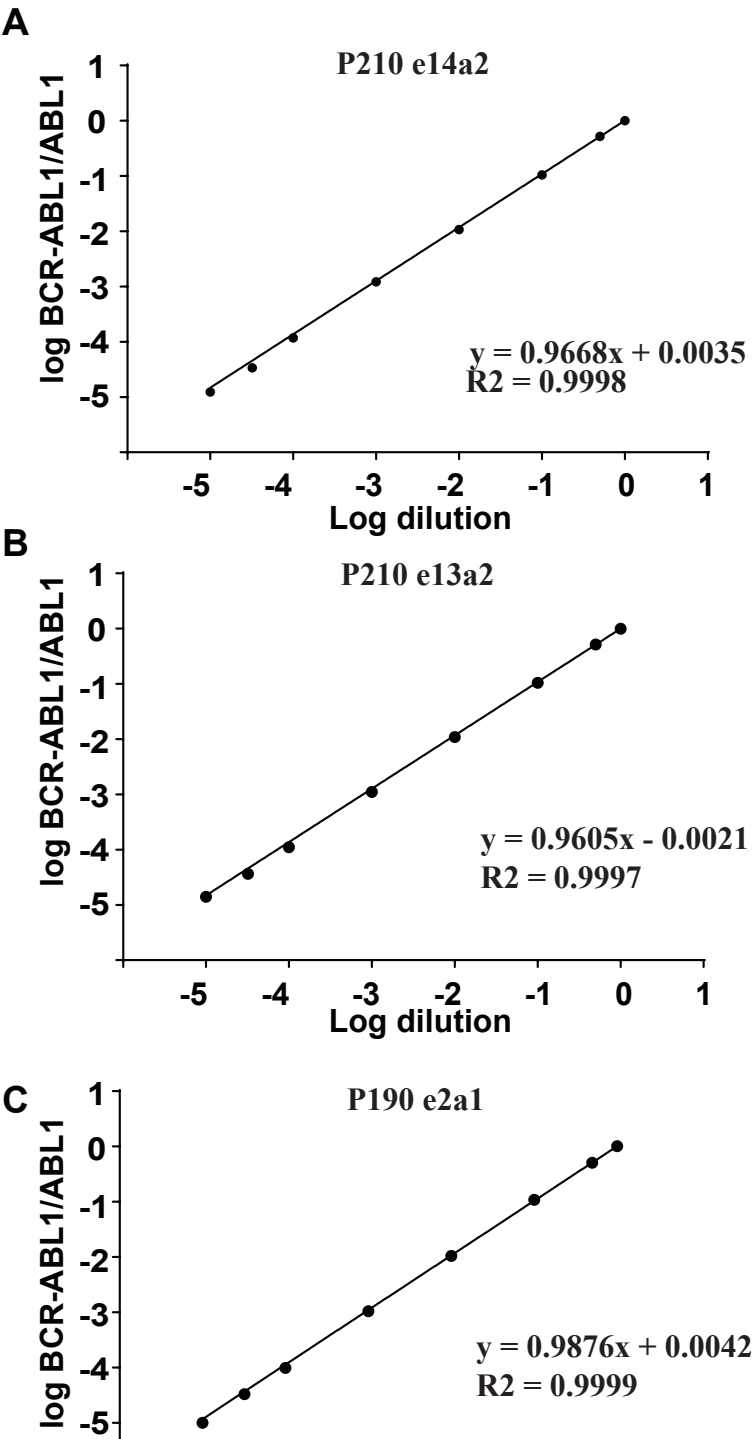

Supplement: Supplementary Figure 1 — Schematic diagram of anti-CD19 CAR-T and anti-CD22 CAR-T. The third generation CAR-T consists of a single-chain variable fragment against CD19 or CD22, two costimulatory domains from CD28 and 4-1BB, and an activation domain (CD3 zeta chain). SP, signal peptide; VL, variable chain; L, linker; VH, variable H chain. [file Image_1.PDF]
