# Supplementary material for: Clinical Utility of Droplet Digital PCR to Monitor BCR-ABL1 Transcripts of Patients With Philadelphia Chromosome–Positive Acute Lymphoblastic Leukemia Post-chimeric Antigen Receptor19/22 T-Cell Cocktail Therapy
Source: Front Oncol. 2021 Apr 7;11:646499. doi: 10.3389/fonc.2021.646499 (PMC8059437; doi:10.3389/fonc.2021.646499)
Supplement: Supplementary Table 1 — Analytical performance parameters of the ddPCR assay. (A) Performance Parameters of the ddPCR P210 (e14a2) Assay by K562 cell lines dilution. (B) Performance Parameters of the ddPCR P210 (e13a2) Assay by P210 (e13a2)-pool. (C) Performance Parameters of the ddPCR P190 (e1a2) Assay by P190 (e1a2)-pool. cDNAs from pretreatment samples pooled [P210(e)-pool, P190 (e1a2)-pool] together with K562 cell lines were diluted in total cDNA from healthy donors (diluent-pool). ddPCR, droplet digital PCR. ddPCR data were analyzed with QuantaSoft analysis software. The target concentration in each sample was expressed as BCR-ABL1 copies/μg. [file Table_1.DOCX]

**Supplementary Table 1. Analytical performance parameters of the ddPCR Assay.**

1. **Performance Parameters of the ddPCR P210 (e14a2) Assay by K562 cell lines dilution.**

| Number of replicates | Dilution series target value (%) | Data mean of BCR-ABL1 fusion (copies/μg) |  |
| --- | --- | --- | --- |
| 4 | 100% (1) | 216000 |  |
| 4 | 50% (0.5) | 110000.0 |  |
| 4 | 10% (10^-1^) | 23316.8 |  |
| 4 | 1% (10^-2^) | 2300.1 |  |
| 4 | 0.1% (10^-3^) | 245.8 |  |
| 4 | 0.032% (3.2 x 10^-4^) | 26.4 |  |
| 4 | 0.01% (10^−4^) | 7.5 |  |
| 4 | 0.001% (10^−5^) | 2.9 |  |
| 10 | 0 | 0 |  |

1. **Performance Parameters of the ddPCR P210 (e13a2) Assay by P210(e13a2)-pool.**

| Number of replicates | Dilution series target value (%) | Data mean of BCR-ABL1 fusion (copies/μg) |  |
| --- | --- | --- | --- |
| 4 | 100% (1) | 202000 |  |
| 4 | 50% (0.5) | 109306.25 |  |
| 4 | 10% (10^-1^) | 21605.6 |  |
| 4 | 1% (10^-2^) | 2334.2 |  |
| 4 | 0.1% (10^-3^) | 246.7 |  |
| 4 | 0.032% (3.2 x 10^-4^) | 23 |  |
| 4 | 0.01% (10^−4^) | 9.2 |  |
| 4 | 0.001% (10^−5^) | 3.3 |  |
| 10 | 0 | 0 |  |

1. **Performance Parameters of the ddPCR P190 (e1a2) Assay by** **P190 (e1a2)-pool.**

| Number of replicates | Dilution series target value (%) | Data mean of BCR-ABL1 fusion (copies/μg) |  |
| --- | --- | --- | --- |
| 4 | 100% (1) | 191000 |  |
| 4 | 50% (0.5) | 94440 |  |
| 4 | 10% (10^-1^) | 19966 |  |
| 4 | 1% (10^-2^) | 1995 |  |
| 4 | 0.1% (10^-3^) | 193.2 |  |
| 4 | 0.032% (3.2 x 10^-4^) | 17.8 |  |
| 4 | 0.01% (10^−4^) | 6.2 |  |
| 4 | 0.001% (10^−5^) | 1.9 |  |
| 10 | 0 | 0 |  |

cDNAs from pretreatment samples pooled ( P210(e)-pool, P190 (e1a2)-pool ) together with K562 cell lines were diluted in total cDNA from healthy donors (diluent-pool). ddPCR, droplet digital PCR. ddPCR data were analyzed with QuantaSoft analysis software. The target concentration in each sample was expressed as BCR-ABL1 copies/μg.
