# Supplementary material for: Clinical Utility of Droplet Digital PCR to Monitor BCR-ABL1 Transcripts of Patients With Philadelphia Chromosome–Positive Acute Lymphoblastic Leukemia Post-chimeric Antigen Receptor19/22 T-Cell Cocktail Therapy
Source: Front Oncol. 2021 Apr 7;11:646499. doi: 10.3389/fonc.2021.646499 (PMC8059437; doi:10.3389/fonc.2021.646499)
Supplement: Supplementary Table 2 — Baseline characteristics of all enrolled patients with Ph+. [file Table_2.DOCX]

**SUPPLEMENTAL TABLES**

**Table S2.** **Baseline characteristics of all enrolled Ph+ patients.**

| Characteristic | Total (n=10) |
| --- | --- |
| Patient gender |  |
| Male | 7 (70%) |
| Female | 30 (3%) |
| Age, y, median (range) | 41.3 (28-48) |
| 16-40 | 3 (30%) |
| 40-60 | 7 (70%) |
| Complex karyotypes |  |
| Yes | 10 (100%) |
| No | 0 (0%) |
| Dose of CART × 10^6^/kg,  median (range) | CD19: 3.46  CD22: 3.59 |
| CD19 CART cell peak copies/ug, median (range) | 182910 (2259-1474359) |
| CD22 CART cell peak copies/ug, median (range) | 105061 (3695-764103) |
| BM tumor burden before CART, median (range) |  |
| <20% | 8 (80%) |
| ≥20% | 2 (20%) |
| WBC count before CART × 10^9^/L, median (range) |  |
| <30 | 9 (90%) |
| ≥30 | 1 (10%) |
| T315I mutation before CART |  |
| Yes | 5 (50%) |
| No | 5 (50%) |
| Types of previous TKIs |  |
| 1 | 4 (40%) |
| 2 or 3 | 6 (60%) |
| Disease status of 3 months post-CART |  |
| MRD− CR | 2 (20%) |
| MRD+ CR | 5 (50%) |
| No CR | 3 (30%) |
| Subsequent transplant |  |
| With allo-HSCT | 4 (40%) |
| No transplant | 6 (60%) |
| Days to bridge into allo-HSCT,  median (range) | 154 (110-210) |
