# Supplementary material for: Clinical Utility of Droplet Digital PCR to Monitor BCR-ABL1 Transcripts of Patients With Philadelphia Chromosome–Positive Acute Lymphoblastic Leukemia Post-chimeric Antigen Receptor19/22 T-Cell Cocktail Therapy
Source: Front Oncol. 2021 Apr 7;11:646499. doi: 10.3389/fonc.2021.646499 (PMC8059437; doi:10.3389/fonc.2021.646499)
Supplement: Supplementary Table 3 — Balanced characteristics of the patients with sequential molecular remission for more than 3 months (SMR3) or not. [file Table_3.DOCX]

**SUPPLEMENTAL TABLES**

|  | **Ph+ ALL after CAR T-cell therapy (n = 10)** | | |
| --- | --- | --- | --- |
| **Characteristic** | **No SMR3 (n = 4)** | **SMR3 (n = 6)** | ***P*** |
| Patient gender |  |  | 0.2000 |
| Male | 4 (100%) | 3 (50%) |  |
| Female | 0 (0%) | 3 (50%) |  |
| Age, y, median (range) | 41.25 (30-48) | 41.33 (28-51) | 1.0000 |
| 16-40 | 1 (25%) | 2 (33.3%) |  |
| 40-60 | 3 (75%) | 4 (66.7%) |  |
| Complex karyotypes |  |  | 1.0000 |
| Yes | 4 (100%) | 6 (100%) |  |
| No | 0 (0%) | 0 (0%) |  |
| Dose of CART × 10^6^/kg, median (range) | CD19: 3.15 (1-6.33)  CD22: 4.40 (2-7.78) | CD19: 3.67 (2-6)  CD22: 3.05 (2-6) |  |
| BM tumor burden before CART, median (range) |  |  |  |
| <20% | 2 (50%) | 6 (100%) | 0.1333 |
| ≥20% | 2 (50%) | 0 (0%) |  |
| WBC count before CART × 10^9^/L, median (range) |  |  |  |
| <30 | 4 (100%) | 5 (100%) | 1.0000 |
| ≥30 | 0 (0%) | 1(100%) |  |
| T315I mutation before CART |  |  |  |
| Yes | 2 (50%) | 3 (50%) | 1.0000 |
| No | 2 (50%) | 3 (50%) |  |
| Types of previous TKIs |  |  | 1.0000 |
| 1 | 2 (50%) | 2 (33.3%) |  |
| 2 or 3 | 2 (50%) | 4 (66.7%) |  |
| Disease status of 3 months post-CART |  |  | 0.0357 |
| MRD− CR | 0 (0%) | 2 (33.3%) |  |
| MRD+ CR | 4 (100%) | 1 (16.7%) |  |
| No CR | 0 (100%) | 3 (50%) |  |
| Subsequent transplant |  |  |  |
| With allo-HSCT | 0 (0 %) | 4 (67 %) | 0.0762 |
| No transplant | 4 (100 %) | 2 (33 %) |  |
| Days to bridge into allo-HSCT, median (range) | 154 (110-210) | - |  |

**Table S3.** **Balanced characteristics of the patients with SMR3 or not.**
